# Fluoride Inside: Syntheses, Structure, and Optical Properties of the *RE*FWO_4_ Representatives with *RE* = Y, Sm–Lu

**DOI:** 10.1021/acs.inorgchem.6c03159

**Published:** 2026-09-03

**Authors:** Benjamin Knies, Priya Pandey, Markus Suta, Ingo Hartenbach

**Affiliations:** † Institute of Inorganic Chemistry, 9149 University of Stuttgart, Pfaffenwaldring 55, Stuttgart 70569, Germany; ‡ Inorganic Photoactive Materials, Institute of Inorganic Chemistry, 98600 Heinrich Heine University Düsseldorf, Universitätsstraße 1, Düsseldorf 40225, Germany

## Abstract

The reactions of WO_3_, *RE*_2_O_3_, and *RE*F_3_ (*RE* = Y, Sm–Lu) with an excess of CsBr as flux lead to the formations of coarse single crystals with the composition *RE*FWO_4_. In the crystal structure (space group *P*1̅; *a* = 471–467, *b* = 617–589, *c* = 694–676 pm; α ≈ 89°, β = 83–81°, γ ≈ 75°; *Z* = 2), 8-fold coordinated *RE* ^3+^ cations are present in the shape of distorted trigonal dodecahedra consisting of five O^2–^ and three F^–^ anions. One of the latter contacts is gradually detached throughout the series from *RE* = Sm to Lu, so that the coordination number of Sm^3+^ in SmFWO_4_ can be considered as eight, while for LuFWO_4_ the coordination number of Lu^3+^ is more accurately described as seven *plus* one. The W^6+^ cations are surrounded by six O^2–^ anions in distorted octahedral shapes, and these polyhedra are fused together via common edges chains running along [100]. Both YFWO_4_ and GdFWO_4_ were activated with Eu^3+^ and Tb^3+^ cations to investigate the usability of these compounds as host materials for luminescence. Besides steady-state excitation and emission spectra, time-resolved measurements at 80 K and at room temperature were performed.

## Graphical abstract

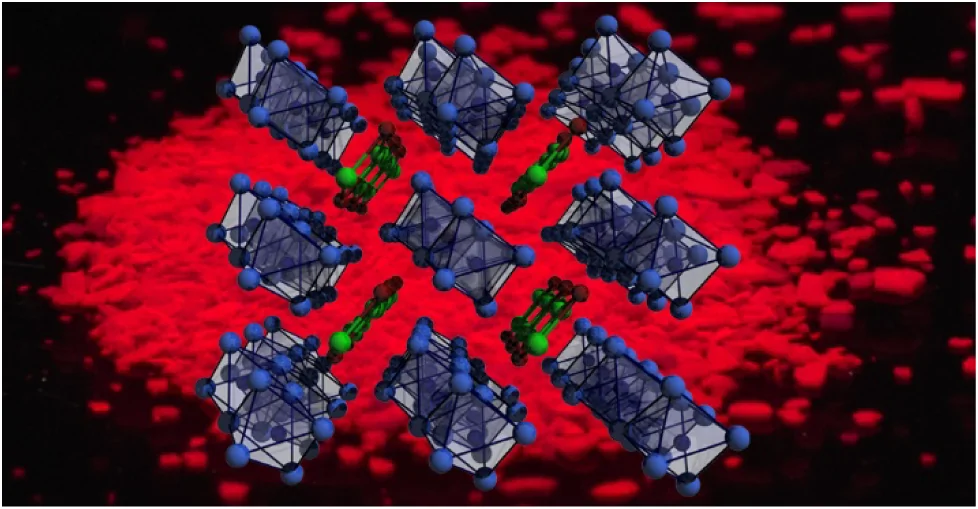

## Introduction

The structural chemistry of rare-earth metal tungstates is very diverse and also rather thoroughly investigated.^1^ These compounds represent interesting host materials for luminescence applications, such as upconversion processes or in lighting.^2^ Halide derivatives of rare-earth metal tungstates, on the other hand, have not experienced such wide-range research interests despite the first representative of this class of compounds, Pr_3_Cl_3_[WO_6_], being known since 1969.^3^ In the early 1980s, the group around Brixner performed thorough investigations of several lanthanum and gadolinium chloride tungstates,^4–10^ and after that another 30-year hiatus of this research topic occurred. While the chloride derivatives with the formula *RE*Cl­[WO_4_] of the larger lanthanoids experience some crystallographic difficulties with respect to the description in the correct unit cell and crystal system,^5^ the representatives of the smaller metals *RE* = Gd–Er crystallize isotypically in the monoclinic system, while those of the smallest *RE* = Tm–Lu also crystallize isotypically in the triclinic system.^11^ Furthermore, bromide derivatives were investigated for the first time with the *RE*Br­[WO_4_] series for the smaller elements *RE* = Y, Gd–Lu again being isotypic to each other in a so far unique triclinic structure.^12^ Some research was also dedicated to the representatives of the larger metals, which unfortunately experience the same crystallographic difficulties as the chloride congeners.^13,14^ All the previously mentioned compounds contain *non*-condensed tetrahedral [WO_4_]^2–^ entities; however, the capacity of hexavalent tungsten to enlarge its coordination number from four to five or six is realized in the *RE*_3_Cl_3_[WO_6_] series,^3,4,7,8,15,16^ in which *non*-condensed trigonal prismatic [WO_6_]^6–^ units are present. While the extension toward iodine derivatives has so far not been successful due to iodide being a strong enough reduction agent to reduce the hexavalent tungsten during the synthesis process, the extension toward the fluoride representatives mostly suffered from side reactions of the F^–^ anions with container materials in solid-state reactions or from poor crystallinity of the obtained products in solvochemical reactions. A first success in synthesizing a fluoride derivative of a rare-earth metal tungstate was achieved by high-pressure synthesis, yielding few single crystals of Gd_5_FW_3_O_16_.^17^ After many more failed attempts of solid-state, hydrothermal, or solvochemical syntheses, the utilization of cesium bromide as flux in high-temperature preparations, which has proven to be successful in synthesizing fluoride-containing rare-earth metal compounds with complex anions, such as *RE*F­[SeO_3_] (RE = Y, Ho–Lu) in the past,^18^ resulted in the title compounds *RE*FWO_4_ (*RE* = Y, Sm–Lu). Thus, the title compounds represent the first series of representatives in the quaternary system RE–W–F–O. Other than for the chloride and bromide derivatives, the rare-earth fluoride tungstates do not crystallize isotypically to their molybdate congeners *RE*F­[MoO_4_] (*RE* = Y,^19^ Ce–Nd,^20^ Sm–Tm^21^) but with a unique structure type and condensed tungstate octahedra instead of tetrahedral [WO_4_]^2–^ units.

## Results and Discussion

### Crystal Structure

The *RE*FWO_4_ representatives with *RE* = Y, Sm–Lu crystallize in the triclinic space group *P*1̅ (no. 2) with two formula units per unit cell and the following ranges of lattice parameters: *a* = 471–467 pm, *b* = 617–589 pm, *c* = 694–676 pm, α ≈ 89°, β = 83–81°, γ ≈ 75°. The unit cell contains one crystallographically unique *RE* ^3+^ site with an 8-fold anionic environment consisting of five oxide and three fluoride anions in a shape best resembling a trigonal dodecahedron (see Figure 1, *left*) according to the program Polynator 1.7 (with deviations from the ideal one in the range of δ = 7.154–8.563 from *RE* = Sm to Lu).^22^

**Figure 1:**
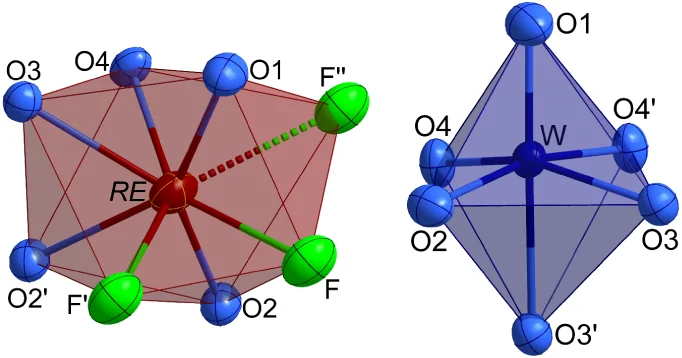
Distorted trigonal [*RE*O_5_F_3_] dodecahedron (*left*) and distorted didigonal [WO_6_] scalenohedron in the crystal structures of the *RE*FWO_4_ representatives with *RE* = Y, Sm–Lu (*right*). Ellipsoid representations at 95% with respect to the YFWO_4_ data.

The interatomic distances within the [*RE*O_5_F_3_] polyhedra are listed in Table 1. They follow the lanthanoid contraction, i.e., becoming smaller throughout the series with the exception of the RE···F″ distance, which increases from SmFWO_4_ to LuFWO_4_ (see Figure 2) due to the diminished coordinative demand of the heavier rare-earth elements. This decrease in the effective coordination number (ECoN)^23^ was also calculated with the program CHARDI,^24,25^ where the F″ ligand exhibits a contribution of 0.72 to the ECoN value of 7.83 for the Sm^3+^ cations in SmFWO_4_, while in LuFWO_4_ the same ligand merely contributes 0.11 to the ECoN value of 7.13 for the Lu^3+^ cations.

**Table 1:** Interatomic Distances (*d*/pm) within the [*RE*O_5_F_3_] Polyhedra Throughout *RE*FWO_4_ Series (*RE* = Y, Sm–Lu)

*RE*	*RE–*O4	*RE–*O1	*RE–*F	*RE–*O2	*RE–*F′	*RE–*O2′	*RE–*O3	*RE···*F″
Y	225.4(2)	227.1(3)	229.3(2)	230.4(2)	231.6(2)	237.4(2)	239.0(2)	264.1(2)
Sm	232.4(4)	234.8(4)	238.2(3)	238.1(4)	241.4(4)	243.6(4)	244.7(4)	255.6(4)
Eu	230.7(3)	233.0(3)	236.3(2)	236.1(3)	240.4(3)	242.0(3)	243.5(3)	255.9(3)
Gd	230.1(3)	232.2(3)	235.1(3)	235.8(3)	239.2(3)	241.6(3)	242.8(3)	256.4(3)
Tb	228.2(3)	230.0(3)	233.0(3)	233.4(3)	237.0(3)	239.6(3)	240.9(3)	258.5(3)
Dy	226.5(2)	228.6(2)	231.3(2)	232.6(2)	234.7(2)	238.9(2)	240.5(2)	260.3(2)
Ho	225.7(3)	227.8(3)	230.4(3)	231.7(3)	233.1(3)	238.2(3)	239.8(3)	262.4(3)
Er	224.7(2)	226.3(2)	228.7(2)	229.2(1)	230.5(2)	236.8(2)	238.0(2)	265.5(2)
Tm	223.7(3)	225.0(3)	227.3(3)	228.1(3)	228.6(3)	235.4(3)	237.0(3)	268.8(3)
Yb	222.4(3)	224.0(3)	225.0(2)	227.1(3)	226.9(3)	234.7(3)	235.5(3)	271.5(3)
Lu	221.9(4)	222.7(4)	224.4(4)	226.2(4)	224.7(4)	233.9(4)	234.7(4)	275.7(4)

**Figure 2:**
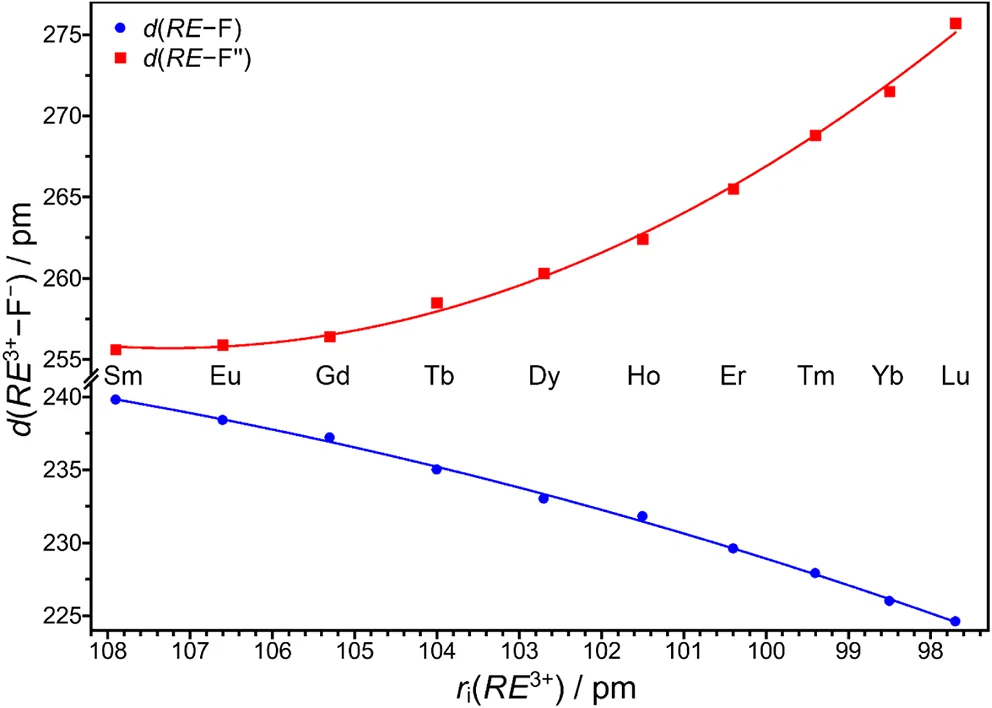
Trend of the interatomic distance between *RE* ^3+^ and the farthest fluoride ligand F″ (red) compared to the distance to the closest F (blue).

The hexavalent tungsten cations are surrounded by six oxygen atoms in a distorted octahedral, or more accurately termed as distorted didigonal scalenohedral fashion (see Figure 1, *right*), according to the program Polynator 1.7 (the deviation from the ideal didigonal scalenohedron ranges about δ = 9.5 ± 0.3 throughout the whole series).^22^ The W^6+^–O^2–^ bond lengths (see Table 2) exhibit the typical behavior found for edge-connected octahedral tungstate entities, i.e., the terminal oxide anions O1 and O2 show the shortest bond lengths, while for the connecting edges O3–O3′ and O4–O4′, one shorter (between 180 and 190 pm) and one longer distance (between 210 and 225 pm) are found. The previously mentioned edge connection leads to {∞1[WO4/2eO2/1t]2−} strands along [100] (see Figure 3, *top*), which remain basically similar-sized throughout the whole series, i.e., they are not affected by the lanthanoid contraction. The partial structure of the *RE* ^3+^ cations and the F^–^ anions can also be described as chains, built up by [*RE*_2_F_2_] rhombi being linked via the longest *RE*–F″ distance to a ladder-like arrangement, running along the crystallographic *a* axis as well (see Figure 3, *bottom*). While the tungstate strand remains spatially rigid throughout the whole series, the shrinking ionic radius of the *RE* ^3+^ cations leads to the *RE*–F″ becoming gradually detached. As a consequence, the *a* lattice parameter does not change significantly from SmFWO_4_ to LuFWO_4_, while the other two steadily decrease (see Figure 4). Both strands are arranged in primitive rod packings, interpenetrating each other to build up the three-dimensional structure (see Figure 5).

**Figure 3:**
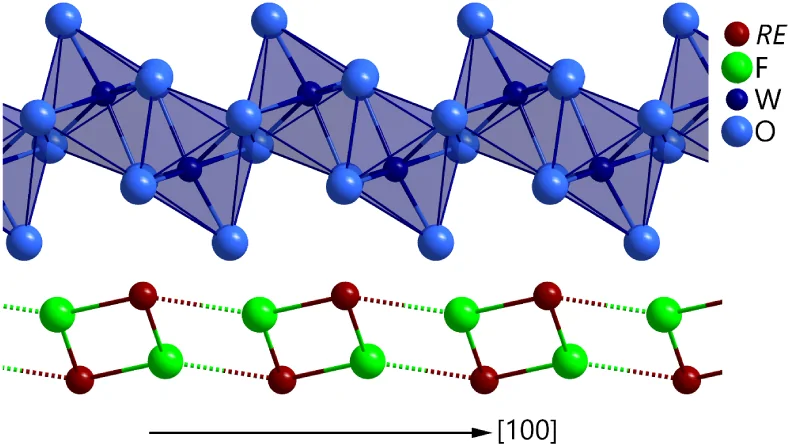
{∞1[WO4/2eO2/1t]2−} strands (*top*) and {∞1[FRE3/3]2+} chains (*bottom*) running along the *a* axis in the structures of the *RE*FWO_4_ representatives with *RE* = Y, Sm–Lu. The longest *RE* ^3+^–F^–^ distances in the latter are displayed in dashed bonds.

**Table 2:** Bond Lengths (*d*/pm) within Tungstate Entities throughout the *RE*FWO_4_ Series (*RE* = Y, Sm–Lu)

*RE*	W*–*O1	W*–*O2	W*–*O3	W*–*O4	W*–*O4′	W*–*O3′
Y	174.0(3)	183.7(2)	184.3(2)	190.0(2)	211.1(2)	224.7(2)
Sm	174.1(4)	183.2(4)	183.8(4)	189.9(4)	210.5(4)	225.9(4)
Eu	174.2(3)	183.9(3)	184.6(3)	190.3(3)	210.3(3)	225.1(3)
Gd	173.6(3)	182.6(3)	184.0(3)	189.8(3)	210.7(3)	225.9(3)
Tb	174.3(3)	184.0(3)	184.3(3)	190.2(3)	210.5(3)	225.3(3)
Dy	173.7(2)	182.8(2)	184.2(2)	190.4(2)	210.3(2)	225.1(2)
Ho	174.0(3)	182.7(3)	184.5(3)	190.1(3)	211.1(3)	224.9(3)
Er	173.9(2)	183.8(2)	185.0(2)	189.9(2)	210.9(2)	224.5(2)
Tm	173.4(3)	183.9(3)	184.6(3)	189.9(3)	211.3(3)	224.4(3)
Yb	173.6(2)	183.2(3)	184.4(3)	190.0(3)	210.9(3)	224.2(2)
Lu	174.0(4)	183.6(4)	184.6(4)	190.2(4)	211.3(4)	224.2(4)

**Figure 4:**
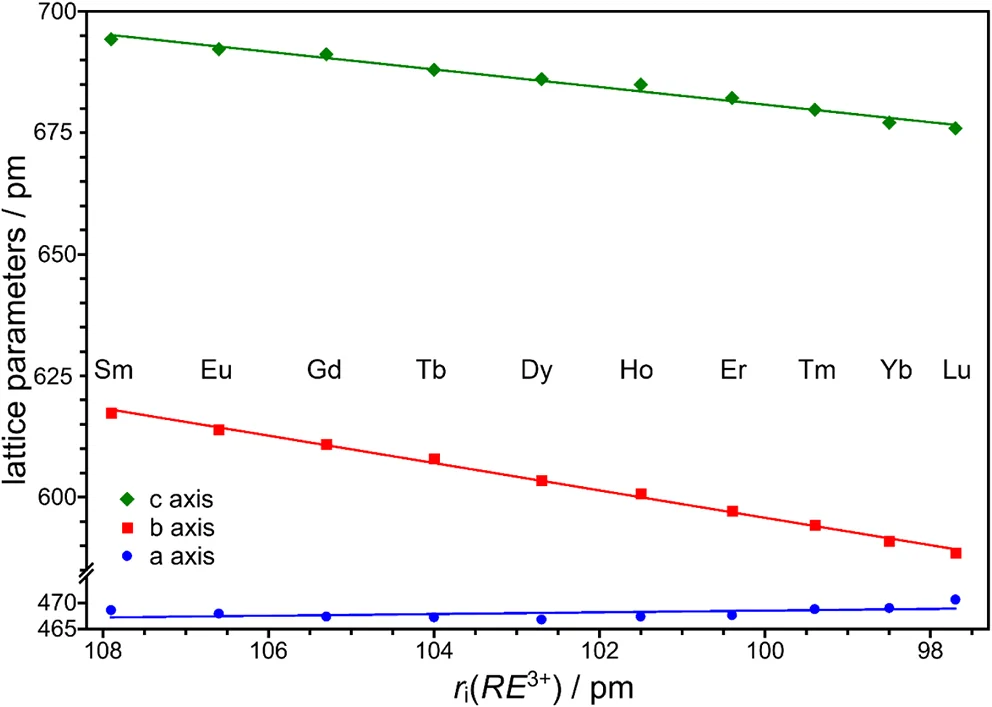
Representation of the unit cell dimensions versus the ionic radius of the respective rare-earth metal cation (for C.N. = 8)^28^ and linear regression of the values. While the *b* and the *c* axes steadily decrease from the Sm to the Lu congener, the *a* axis remains basically constant, exhibiting a minor upward trend.

**Figure 5:**
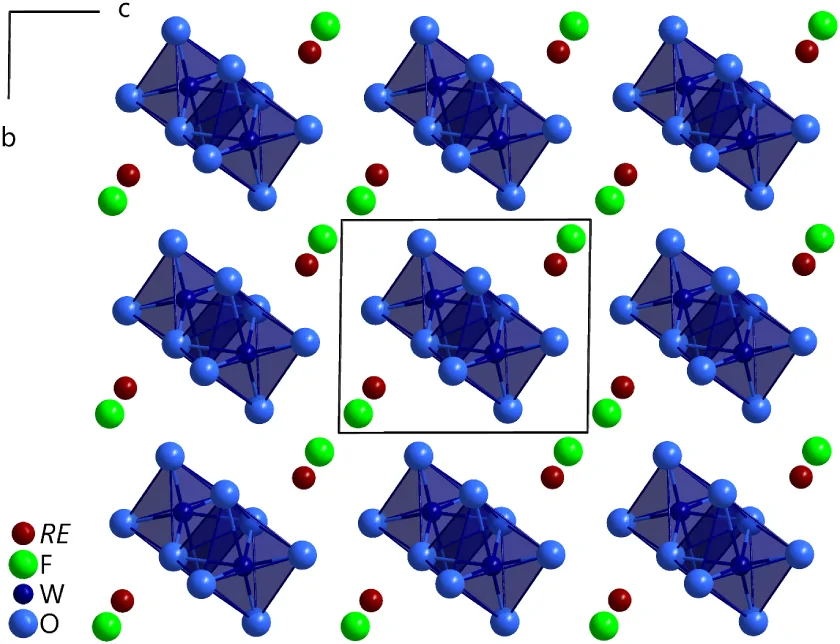
View at the crystal structure of the *RE*FWO_4_ representatives with *RE* = Y, Sm–Lu along [100], emphasizing the interpenetrating rod packing of the {∞1[WO4/2eO2/1t]2−} strands and the {∞1[FRE3/3]2+} chains.

Distinguishing fluoride from oxide anions by means of single-crystal X-ray diffraction is not always unambiguous due to their similar scattering powers. Due to electroneutrality reasons, the F:O ratio of 1:4 has to be maintained in the compounds; however, the question of the correct allocation of the elements to their reported position remains. Two of the oxygen atoms surrounding the W^6+^ cations serve in an interconnecting capacity; thus, due to chemical reasons, it is highly unlikely that these oxide anions would be even partially substituted by fluoride. Attempts to refine the crystal structures with a partial or complete fluoride/oxide exchange on the remaining positions F, O1, and O2, respectively, result in significantly higher residual values as well as in too low (for refining oxygen on the fluoride position) or rather high anisotropic displacement parameters (for refining fluorine on the O1 or the O2 position), respectively. This was also observed during the refinement of the respective rare-earth-metal-fluoride molybdates *RE*F­[MoO_4_] ^19–21^ and REFMo_2_O_7_.^26,27^ Furthermore, bond-valence calculations with the program CHARDI result in a 300 to 600% higher deviation from the ideal ionic charges if the F and the O1 (or O2) positions are interchanged.^24,25^

### Single-Crystal Raman Spectroscopy

Single-crystal Raman spectra were acquired for all crystals. Since all structures are isotypic, Figure 6 shows the spectrum of SmFWO_4_ as representative of the whole series. The highest frequency peak in the spectrum is located at about 946 cm^–1^, which correlates to stretching vibrations of the terminal W^6+^–O^2–^ groups.^29^ Stretching vibrations as well as deformation modes relating to the longer edge-connecting W^6+^–O^2–^ entities are found from 790 down to 500 cm^–1^, while the bands at lower wavenumbers are assigned to *RE* ^3+^–O^2–^/F^–^ as well as lattice vibrations.

**Figure 6:**
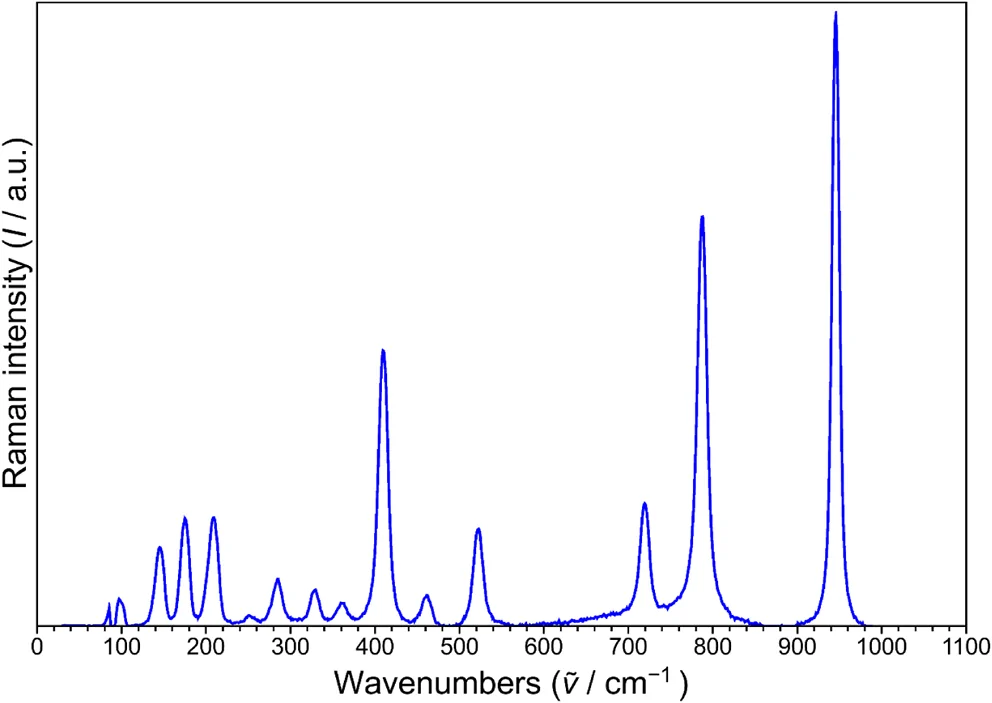
Single-crystal Raman spectrum of SmFWO_4_ as a typical representative of the *RE*FWO_4_ series with *RE* = Y, Sm–Lu.

### Luminescence Spectroscopy

YFWO_4_ and GdFWO_4_ were activated with nominally 1 mol % Eu^3+^ and Tb^3+^, respectively, to investigate their luminescence properties. The excitation and emission spectra at *T* = 80 K are depicted in Figures 7 and 8. Both compounds show the typical 4f^6^ ← 4f^6^ of Eu^3+^ or 4f^8^ ← 4f^8^ transitions of Tb^3+^ in their excitation spectra, respectively. In addition, broad excitation bands are observable in the UV range that probably stem from a W^6+^ ← O^2–^ ligand-to-metal charge transfer (LMCT) transition (eventually with overlapping 4f^7^5d^1^ ← 4f^8^ transitions in the case of the Tb^3+^-activated compounds that cannot be fully excluded in that spectral range). At 80 K, the intensity of those bands is comparably low, which indicates a limited indirect crossover *via* these states.

**Figure 7:**
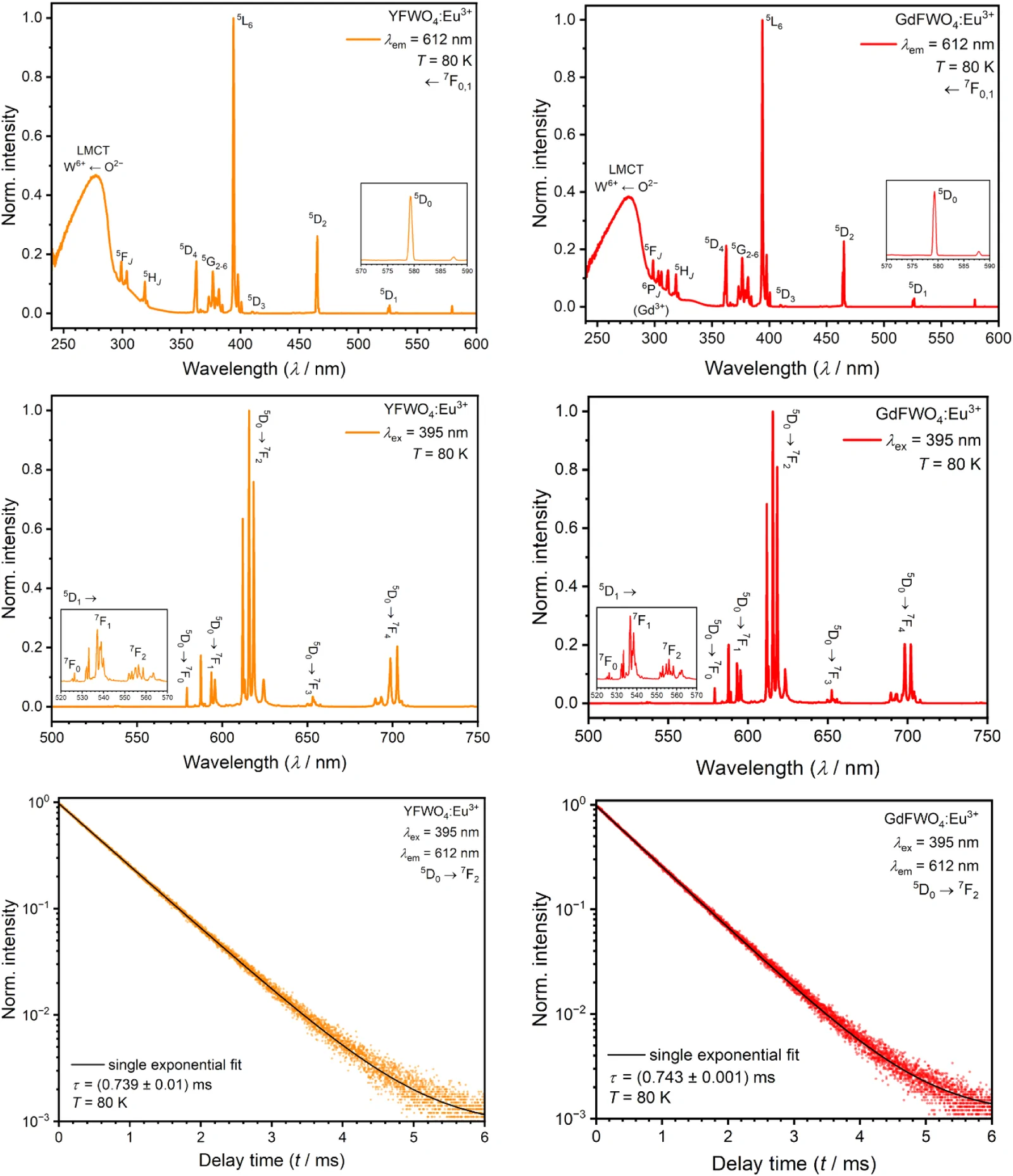
Excitation (*top*) and emission spectra (*middle*) of YFWO_4_:Eu^3+^ (*left column, orange graphs*) and GdFWO_4_:Eu^3+^ (*right column, red graphs*) at *T* = 80 K. Decay curves of the ^5^D_0_-based luminescence at *T* = 80 K are shown at the *bottom*.

**Figure 8:**
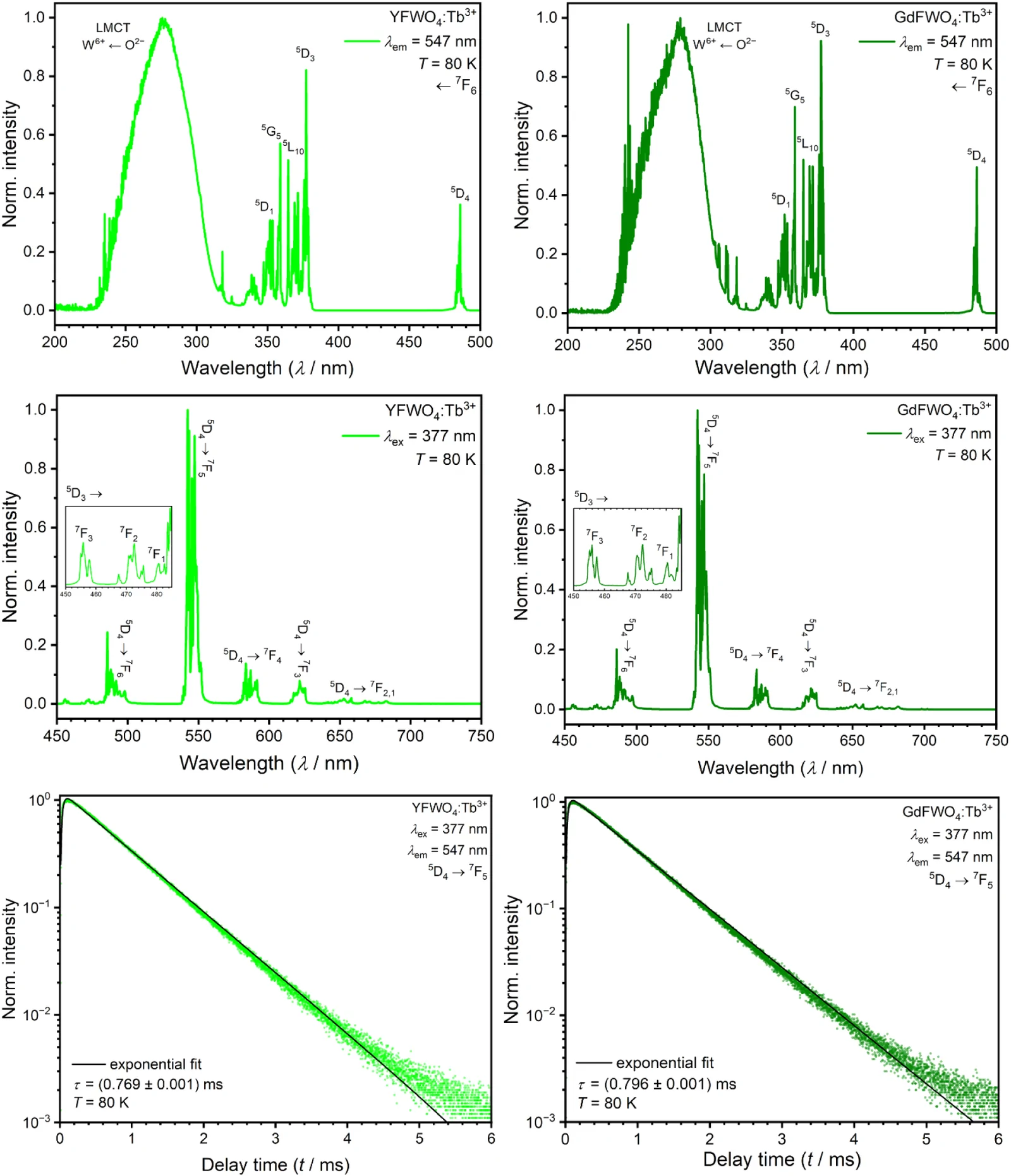
Excitation (*top*) and emission spectra (*middle*) of YFWO_4_:Tb^3+^ (*left column, bright green graphs*) and GdFWO_4_:Tb^3+^ (*right column, dark green graphs*) at *T* = 80 K. Decay curves of the ^5^D_4_-based luminescence at *T* = 80 K are shown at the *bottom*.

Only a single line for the ^5^D_0_ ↔ ^7^F_0_ transition is observed, which indicates only one crystallographically independent *RE* ^3+^ site in the regarded fluoride tungstates, in agreement with the implications from the single crystal structure data. The emission spectra of the Eu^3+^-activated fluoride tungstates are dominated by transitions from the ^5^D_0_ level, even upon excitation into the ^5^L_6_ level at 396 nm. This is surprising given the correspondingly low cutoff phonon energy of 946 cm^–1^ (see Figure 6) that would formally imply rather slow nonradiative decay from the ^5^D_1_ level (Δ*E* = 1750 cm^–1^) according to the energy gap law. In fact, the total decay time of the ^5^D_1_ level in these hosts is merely between 7 and 8 μs. This is to be compared to, e.g., YAl_3_[BO_3_]_4_:Eu^3+^ with a higher cutoff phonon energy of around 1400 cm^–1^ ^30^ but yet a corresponding decay time of the ^5^D_1_ level of almost 18 μs,^31^ in contrast to the expectations of the energy gap law. The observation of dominant ^5^D_0_-based luminescence indicates that nonradiative multiphonon relaxation in the fluoride tungstate is unusually fast, which will be investigated in more detail in a future dedicated study. The decay time of τ = 0.77–0.79 ms of the ^5^D_0_ level (single exponential decay) at *T* = 80 K is in line with that low cutoff phonon energy but unusually short and indicates a strongly induced electric dipolar character of the respective transition. This observation also agrees with the strong hypersensitive ^5^D_0_ → ^7^F_2_ transition at 613 nm that is particularly affected by this admixture according to Judd–Ofelt theory. Again, the comparison to YAl_3_[BO_3_]_4_:Eu^3+^ is insightful here, as the corresponding decay time of the ^5^D_0_ level in that compound is longer with τ = 1.35 ms despite its higher cutoff phonon energy.^31^ In the case of the Tb^3+^-activated fluoride tungstates, the emission spectra show dominant, typical green luminescence from the energetically isolated ^5^D_4_ level of Tb^3+^ with a similarly short decay time of τ = 0.75 ms at *T* = 80 K despite the low maximum vibrational energy. To compare, the respective decay time in the huntite-type borate YAl_3_[BO_3_]_4_:Tb^3+^ at *T* = 80 K is around τ = 2.0 ms,^32^ again in contrast to expectations according to the energy gap law. This strong difference also implies a strong admixture of the LMCT state into the 4f^8^ spin–orbit levels of Tb^3+^ in the regarded fluoride tungstates. Interestingly, excitation into the higher energetic ^5^D_3_ level reveals radiative ^5^D_3_ → ^7^F_*J*_ (*J* = 6···0) transitions in the blue spectral range. This is to be expected given the very large energy gap of around Δ*E* = 5700 cm^–1^ between the ^5^D_3_ and ^5^D_4_ levels compared to the maximum phonon energy of 946 cm^–1^ (see Figure 6) that basically limits nonradiative multiphonon relaxation. Yet, the relative intensity of the ^5^D_3_-based emission below 500 nm is unusually small given those boundary conditions. One possible reason could be cross relaxation of the ^5^D_3_ level, which can be, however, limited at low Tb^3+^ fractions. The closest *RE*–*RE* distances in the regarded fluoride tungstates are 375–385 pm (*RE* = Y) or 387–393 pm (*RE* = Gd), which makes such an energy transfer process probable in principle. However, such energy transfer processes should lead to multiexponential decays,^33,34^ which is not observed in this sample probing the time-resolved luminescence of the ^5^D_3_ level with a total decay time of τ = 0.13 ms at *T* = 80 K. Like in the case of Eu^3+^, the corresponding decay time of the ^5^D_3_ level in YAl_3_[BO_3_]_4_:Tb^3+^ at *T* = 80 K is τ = 0.03 ms, which is surprisingly similar despite the lower number of around *p* = 4 required phonon modes compared to *p* = 6 for *RE*FWO_4_:Tb^3+^ (*RE* = Y, Gd). Thus, also for Tb^3+^, an unusually fast nonradiative decay based on admixture of the energetically close LMCT state may be anticipated that will be investigated in more detail in a future study.

### Measurements of Magnetic Properties

The shielded nature of the 4f orbitals generally limits exchange interactions and thus typically leads to ordering temperatures of well below 10 K in the case of rare-earth compounds. Together with the shortest distances between the solely magnetically active cations Gd^3+^ in the investigated sample of GdFWO_4_ being 386 pm, magnetic interactions between them are highly unlikely. Thus, the sample displays almost ideal Curie–Weiss behavior throughout the entire temperature range (see Figure 9). The respective fit according to χ = *C*/(*T* – θ) revealed a Curie constant of *C* = 7.53(1) K and θ = −0.61(1) K, which results in a calculated magnetic moment of 7.76(1) μ_B_. This is about 2% slightly smaller than the theoretical one of 7.94 for Gd^3+^ cations, which can probably be explained by a slight amount of amorphous, nonmagnetic byproduct, such as SiO_2_ from the reaction container.

**Figure 9:**
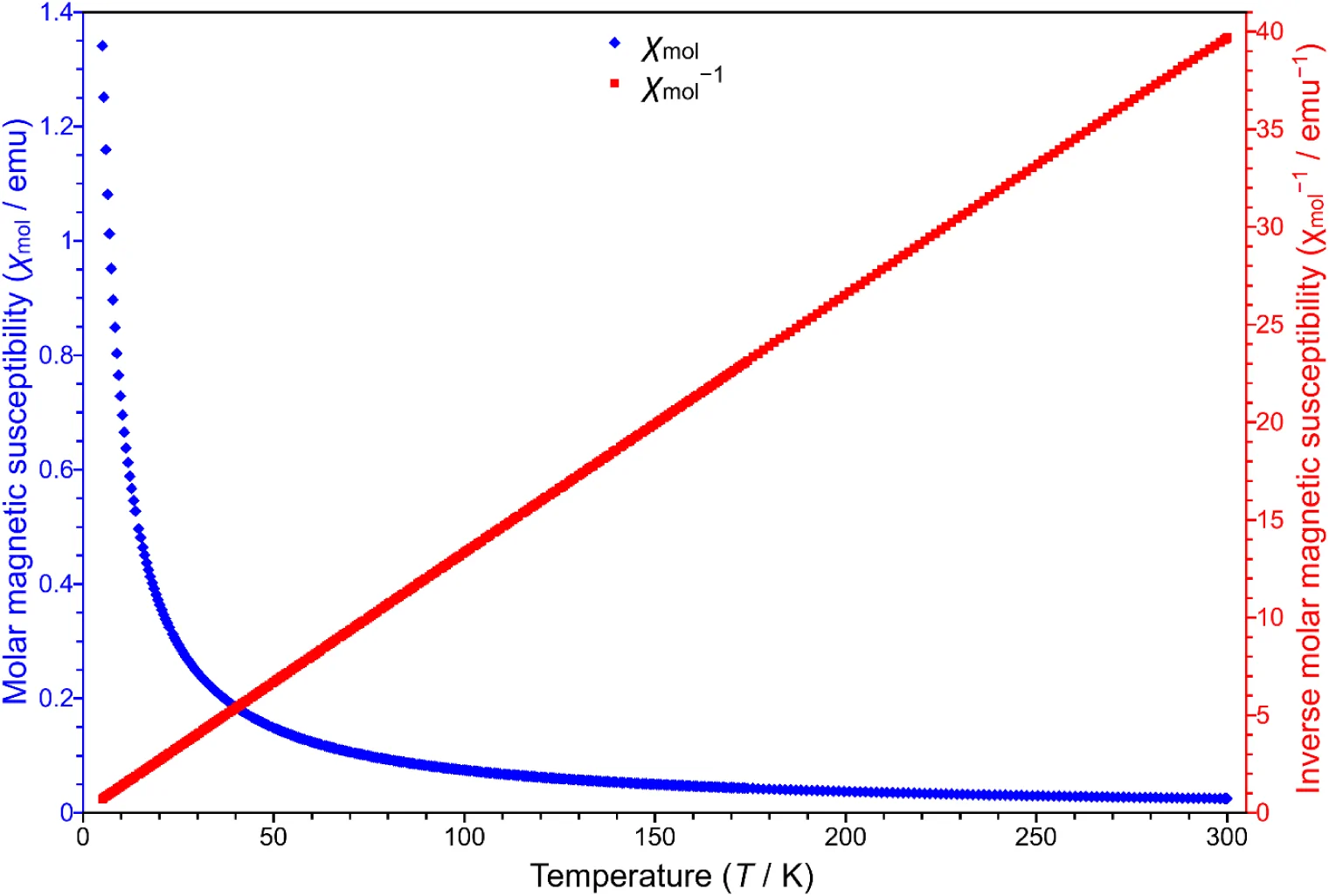
Molar magnetic susceptibility (χ_mol_, *blue curve*) and inverse molar magnetic susceptibility (1/χ_mol_, *red curve*) of GdFWO_4_.

## Conclusion

In this publication, the discovery of the novel rare-earth metal fluoride oxidotungstates with the composition *RE*FWO_4_ for the smaller elements *RE* = Y, Sm–Lu is reported. After countless synthesis attempts, the utilization of cesium bromide as a fluxing agent proved to be successful in producing this class of compound. Besides the crystal structure determination, the luminescence properties of Eu^3+^- and Tb^3+^-doped yttrium and gadolinium representatives were investigated. In all cases, the typical W^6+^ ← O^2–^ ligand-to-metal charge transfer is visible with a maximum of 285 nm, which is at wavelengths usually expected for hexavalent tungsten in a tetrahedral coordination.^35^

## Experimental Section

### Syntheses

0.25 mmol of the rare-earth metal oxides *RE*_2_O_3_, namely Y_2_O_3_ (99.9%, ChemPur, Karlsruhe, Germany), Sm_2_O_3_ (99.9%, ChemPur, Karlsruhe, Germany), Eu_2_O_3_ (99.99%, ChemPur, Karlsruhe, Germany), Gd_2_O_3_ (99.99%, Aldrich, Darmstadt, Germany), Dy_2_O_3_ (99.9%, ChemPur, Karlsruhe, Germany), Ho_2_O_3_ (99.9%, ChemPur, Karlsruhe, Germany), Er_2_O_3_ (99.999%, ChemPur, Karlsruhe, Germany), Tm_2_O_3_ (99.9%, ChemPur, Karlsruhe, Germany), Yb_2_O_3_ (99.99%, ChemPur, Karlsruhe, Germany), Lu_2_O_3_ (99.9%, ChemPur, Karlsruhe, Germany) and the same amount of rare-earth metal fluorides *RE*F_3_, YF_3_ (99.9%, ChemPur, Karlsruhe, Germany), SmF_3_ (99.9%, Heraeus, Hanau, Germany), EuF_3_ (99.9%, ChemPur, Karlsruhe, Germany), GdF_3_ (99.99%, Aldrich, Darmstadt, Germany), TbF_3_ (99.9%, ChemPur, Karlsruhe, Germany), DyF_3_ (99.9%, ChemPur, Karlsruhe, Germany), HoF_3_ (99%, ChemPur, Karlsruhe, Germany), ErF_3_ (99.9%, ChemPur, Karlsruhe, Germany), TmF_3_ (99.9%, ChemPur, Karlsruhe, Germany), YbF_3_ (99.9%, ChemPur, Karlsruhe, Germany), LuF_3_: (99.9%, ChemPur, Karlsruhe, Germany), as well as 0.75 mmol tungsten trioxide WO_3_ (99.9%, Aldrich, Darmstadt, Germany) were heated together with 800 mg CsBr (99%, abcr, Karlsruhe, Germany) as flux in fused and graphitized, evacuated silica ampules for 7 days at 700 °C. Due to the commercial unavailability of Tb_2_O_3_, a 3:2 mixture of Tb_4_O_7_ (99.9%, Onyxmet, Olsztyn, Poland) and 11 mg Tb (99.9%, ChemPur, Karlsruhe, Germany) was used for the in situ generation of Tb_2_O_3_. The furnace was cooled down in 2 days to 630 °C and then in 1 day to room temperature. The ampules were opened and the contained bulk product, which remains stable to atmospheric influences, was washed with demineralized water and was dried at 50 °C. Finally, coarse single crystals, exhibiting the color of the respective trivalent rare-earth metal cation (colorless for *RE* = Y, Eu, Gd, Tb, Dy, Tm, Yb, Lu; pale yellow for *RE* = Sm; yellow for RE = Ho; and pink for *RE* = Er), were selected for X-ray diffraction and Raman spectroscopy. For the synthesis of the activated samples of YFWO_4_ and GdFWO_4_, double the amount of the initial reaction mixture was used with an activation grade of about 1% Eu_2_O_3_ or Tb_4_O_7_, respectively.

### Single Crystal Structure Determination

Single crystal data sets of the *RE*FWO_4_ representatives were collected with the help of a StadiVari diffractometer (Stoe & Cie., Darmstadt, Germany) equipped with a microfocus source emitting Ag–K_α_ radiation (λ = 56.093 pm) and a Pilatus 200K detector (Dectris, Baden, Switzerland). Background, polarizations, and Lorentz factor corrections as well as scaling of the reflection intensities were performed by the program STOE LANA.^36^ Structure solutions and refinements were carried out with the program package SHELX-2019 ^37,38^ (65 refined parameters for all crystals). The compounds crystallize isotypically in the triclinic space group *P*1̅ with two formula units per unit cell. For some of the structure refinements, a high residual electron density is observed around the tungsten and/or the rare-earth metal atoms in the structure. This is mostly due to the rather high diffraction angle, which produces these artifacts. However, due to refining very strong diffracting atoms (W and *RE*) next to rather weak ones (O and F), the high angle is needed in order to gain enough information on the latter ones for adequate positional and anisotropic refinement. Cutting those angles to significantly smaller ones would result in less residual electron density; however, it would also result in the loss of the ability to refine oxygen and fluorine anisotropically. Further information on the crystallographic data can be found in Tables 3–5 as well as from the joint CCDC/FIZ-Karlsruhe online deposition service: https://www.ccdc.cam.ac.uk/structures/? by quoting the deposition numbers CSD-2456963 for YFWO_4_, CSD-2456971 for SmFWO_4_, CSD-2456969 for EuFWO_4_, CSD-2456970 for GdFWO_4_, CSD-2456964 for TbFWO_4_, CSD-2456973 for DyFWO_4_, CSD-2456966 for HoFWO_4_, CSD-2456967 for ErFWO_4_, CSD-2456968 for TmFWO_4_, CSD-2456972 for YbFWO_4_, CSD-2456965 for LuFWO_4_.

**Table 3:** Crystallographic Data of the *RE*FWO_4_ Representatives with *RE* = Y, Sm, Eu, and Gd

Crystallographic data	YFWO_4_	SmFWO_4_	EuFWO_4_	GdFWO_4_
*a*/pm	467.26(2)	468.55(3)	467.88(1)	467.33(2)
*b*/pm	599.66(3)	617.35(4)	613.88(1)	610.95(3)
*c*/pm	683.55(3)	694.31(4)	692.24(2)	691.19(4)
α/°	88.879(4)	89.497(5)	89.367(2)	89.271(4)
β/°	82.256(4)	83.149(5)	83.030(2)	82.955(4)
γ/°	75.227(4)	74.839(5)	74.892(2)	74.940(4)
ρ_c_/g·cm^–1^	6.439	7.201	7.302	7.449
*V*_m_/cm^3^·mol^–1^	55.250	57.936	57.358	56.936
*F*(000)	308	354	356	358
Measuring range ±*h*; ±*k*; ±*l*	7; –7/9; 11	7; 10; –9/11	7; –10/8; 11	7; 10; –10/11
2θ_max_/°	55.71	56.74	55.72	55.71
μ/mm^–1^	25.40	24.10	24.87	25.62
Number of coll./unique reflections	8176/1682	11047/1959	9629/1848	8363/1784
*R*_int_/*R*_σ_	0.018/0.018	0.041/0.045	0.023/0.015	0.020/0.019
*R*_1_ for *n* refl. with |*F*_0_| ≥ 4σ_o_(*F*_0_)	0.016; *n* = 1622	0.022; *n* = 1609	0.022; *n* = 1802	0.020; *n* = 1690
*R*_1_/*wR*_2_ for all reflections	0.017/0.038	0.030/0.049	0.022/0.054	0.021/0.050
*GooF*	1.084	0.945	1.087	1.079
Residual electron density ρ_max_/ρ_min_/10^–6^ *e* ^–^·pm^–3^	1.93/–1.85	3.11/–3.23	2.25/–3.92	2.47/–2.44

**4:** Crystallographic Data of the *RE*FWO_4_ Representatives with *RE* = Tb, Dy, Ho, and Er

Crystallographic data	TbFWO_4_	DyFWO_4_	HoFWO_4_	ErFWO_4_
*a*/pm	467.19(1)	466.78(3)	467.33(2)	467.59(2)
*b*/pm	607.98(2)	603.45(3)	600.78(3)	591.17(2)
*c*/pm	688.01(2)	686.08(4)	684.20(3)	682.19(3)
α/°	89.028(2)	89.000(4)	88.898(3)	88.836(3)
β/°	82.606(2)	82.482(4)	82.283(3)	82.127(3)
γ/°	74.935(2)	75.096(4)	75.183(3)	75.328(3)
ρ_c_/g·cm^–1^	7.557	7.703	7.785	7.899
*V*_m_/cm^3^·mol^–1^	56.340	55.741	55.464	54.958
*F*(000)	360	362	364	366
Measuring range ±*h*; ±*k*; ±*l*	7; –6/10; 11	7; –10/9; –10/11	7; –10/8; 11	–6/7; 9; 11
2θ_max_/°	55.72	55.72	55.73	55.72
μ/mm^–1^	26.48	27.39	28.12	29.15
Number of coll./unique reflections	10409/1812	11906/1791	11621/1780	10009/1758
*R*_int_/*R*_σ_	0.026/0.020	0.019/0.017	0.032/0.022	0.018/0.013
*R*_1_ for *n* refl. with |*F*_0_| ≥ 4σ_o_(*F*_0_)	0.022; *n* = 1663	0.013; *n* = 1604	0.021; *n* = 1631	0.015; *n* = 1699
*R*_1_/*wR*_2_ for all reflections	0.025/0.056	0.016/0.026	0.024/0.050	0.016/0.037
*GooF*	0.998	1.004	1.102	1.089
Residual electron density ρ_max_/ρ_min_/10^–6^ *e* ^–^·pm^–3^	3.63/–3.24	1.46/–1.37	2.43/–2.36	1.41/–1.46

**Table 5:** Crystallographic Data of the *RE*FWO_4_ Representatives with *RE* = Tm, Yb, and Lu

Crystallographic data	TmFWO_4_	YbFWO_4_	LuFWO_4_
*a*/pm	468.76(2)	468.96(2)	470.61(2)
*b*/pm	594.26(3)	590.99(3)	588.58(3)
*c*/pm	679.78(3)	677.08(3)	675.93(3)
α/°	88.778(4)	88.777(4)	88.750(4)
β/°	81.820(3)	81.652(4)	81.411(4)
γ/°	75.337(3)	75.498(4)	75.360(4)
ρ_c_/g·cm^–1^	7.982	8.128	8.183
*V*_m_/cm^3^·mol^–1^	54.594	54.118	53.992
*F*(000)	368	370	372
Measuring range ±*h*; ±*k*; ±*l*	7; 9; 11	7; –9/8; 11	–7/5; 9; 11
2θ_max_/°	55.72	55.71	55.73
μ/mm^–1^	30.00	31.05	31.90
Number of coll./unique reflections	9549/1747	10434/1736	11814/1736
*R*_int_/*R*_σ_	0.027/0.017	0.027/0.024	0.039/0.025
*R*_1_ for *n* refl. with |*F*_0_| ≥ 4σ_o_(*F*_0_)	0.022; *n* = 1687	0.017; *n* = 1516	0.024; *n* = 1586
*R*_1_/*wR*_2_ for all reflections	0.023/0.056	0.021/0.036	0.028/0.060
*GooF*	1.166	0.966	1.083
Residual electron density ρ_max_/ρ_min_/10^–6^ *e* ^–^·pm^–3^	3.34/–4.15	1.49/–1.76	4.25/–3.53

### X-ray Powder Diffractometry

Powder X-ray data were collected for all compounds on a STADI-P powder diffractometer (Stoe & Cie., Darmstadt, Germany) equipped with a MYTHEN-1K detector (Dectris, Baden, Switzerland), utilizing monochromatized Cu–K_α1_ radiation (λ = 154.056 pm). The samples were recorded in transmission mode upon placing the bulk product on appropriate adhesive tape. In most cases, the bulk products could not be confirmed to be single phase due to the reaction in silica ampules with rare-earth metal fluorides as reactants, which are known to attack the container walls, even if they are graphitized. A LeBail-Fit, performed with the program package FullProf,^39^ of YFWO_4_ as a representative for the whole series, is depicted in Figure 10. The rather ragged difference curve (blue line) can be attributed to the uneven texture of the crystallites.

**Figure 10:**
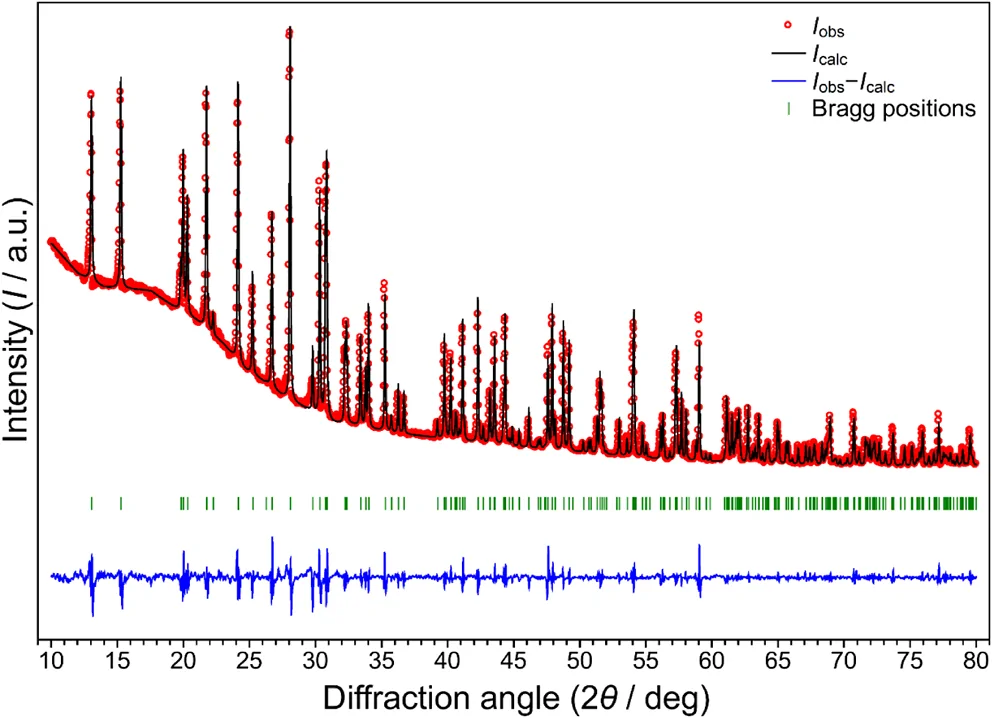
LeBail fit of the powder pattern of YFWO_4_.

### Raman Spectroscopy

Single-crystal Raman spectra of the title compounds were collected with the help of a Horiba XploRa spectrometer (Kyoto, Japan), utilizing a red LASER with a wavelength of λ = 632 nm.

### Luminescence Spectroscopy

Photoluminescence spectra were acquired with an FLS1000 photoluminescence spectrometer from Edinburgh Instruments. It was equipped with a 450 W Xe arc lamp for excitation, double grating excitation, and emission monochromators in Czerny-Turner configuration, and a thermoelectrically cooled (−20 °C) photomultiplier tube PMT-980 (Hamamatsu). Emission spectra were corrected with respect to the grating efficiency and PMT sensitivity, while excitation spectra were additionally corrected with respect to the lamp intensity. Time-resolved luminescence was probed with an Edinburgh Instruments μF2 μs flash lamp (60 W pulsed Xe arc lamp) with adjustable trigger frequency (0.1 Hz···1000 Hz) and temporal pulse width of Δτ ≈ 2 μs using single-photon multichannel scaling (MCS) as the detection mode. Low-temperature measurements were performed with a Linkam Scientific THMS600 temperature cell with an accuracy of ±0.1 °C in the temperature range between −196 °C (liquid nitrogen) and room temperature regarded within this work. The signal was coupled to the spectrometer with optical fiber bundles from the temperature cell.

### Measurements of Magnetic Properties

54 mg of microcrystalline GdFWO_4_ was placed in a gelatin capsule and attached to the sample holder of a vibrating sample magnetometer for measuring the magnetization *M*(*T*) in a magnetic property measurement system (MPMS3, Quantum Design, San Diego, CA, USA). The sample was examined in the temperature range between 5 and 300 K in a homogeneous magnetic field of 500 Oe to calculate the molar (χ_mol_) and the inverse molar susceptibility (1/χ_mol_) of the aforementioned sample. Diamagnetic corrections were performed according to Bain and Berry.^40^

## References

[1] Schleid T., Hartenbach I. (2016). On Halide Derivatives of Rare-Earth Metal­(III) Oxidomolybdates­(VI)
and -tungstates­(VI). Z. Kristallogr..

[2] Kaczmarek A. M., van Deun R. (2013). Rare-earth tungstate
and molybdate compounds –
from 0D to 3D architectures. Chem. Soc. Rev..

[3] Polyanskaya T. M., Borisov S. V., Belov N. V. (1969). The Crystal
Structure of Pr_3_WO_6_Cl_3_. Dokl. Akad. Nauk
SSSR.

[4] Brixner L.
H., Chen H.-Y., Foris C. M. (1982). Structure and Luminescence of Some
Rare-Earth Halotungstates of the type Ln_3_WO_6_Cl_3_. J. Solid State Chem..

[5] Brixner L. H., Chen H.-Y., Foris C. M. (1982). Structure
and Luminescence of the
Orthorhombic LnWO_4_Cl-Type Rare-Earth Halo Tungstates. J. Solid State Chem..

[6] Brixner L. H., Chen H.-Y., Foris C. M. (1982). Structure and Luminescence
of the
Monoclinic LnWO_4_Cl-type Rare-Earth Halotungstates. Mater. Res. Bull..

[7] Blasse G., Dirksen B. J., Brixner L. H. (1982). Luminescence Properties
of Uranium-Activated
Lanthanum Halotungstate (La_3_WO_6_Cl_3_-U). J. Solid State Chem..

[8] Parise J. B., Brixner L. H., Prince E. (1983). Refinement
of the Structure of Trilanthanum
Trichlorohexaoxotungstate, La_3_WO_6_Cl_3_, from Neutron Powder Diffraction Data. Acta
Crystallogr..

[9] Blasse G., Bokkers G., Dirksen B. J., Brixner L. H. (1983). Luminescence in
Lanthanum Chlorotunstate. J. Solid State Chem..

[10] Blasse G., Brixner L. H. (1983). Luminescence in
Gadoliniumchlorotungstate (GdWO_4_Cl). J. Solid State Chem..

[11] Schustereit T., Schleid T., Höppe H. A., Kazmierczak K., Hartenbach I. (2015). Chloride Derivatives of Lanthanoid­(III) *Ortho*-Oxidotungstates­(VI) with the Formula *Ln*Cl­[WO_4_] (*Ln* = Gd - Lu): Syntheses, Crystal
Structures
and Spectroscopic Properties. J. Solid State
Chem..

[12] Schustereit T., Schleid T., Hartenbach I. (2015). Syntheses and Crystal Structures
of the Rare-earth Metal­(III) Bromide *Ortho*-Oxidotungstates­(VI)
with the Formula *RE*Br­[WO_4_] (*RE* = Y, Gd-Yb). Solid State Sci..

[13] Jiao Z., Mireles O. M., Ensz K., Wang F., Liang M., Halasyamani P. S., Zhang B., Rillema D. P., Wang J. (2023). Heteroanionic
LaBrVIO_4_ (VI = Mo, W): excellence in both nonlinear optical
properties and photoluminescent properties. Chem. Mater..

[14] Jiao Z., Quah J., Syed T. H., Wei W., Zhang B., Wang F., Wang J. (2024). Synthesis, crystal
and electronic
structures, linear and nonlinear optical properties, and photocurrent
response of oxyhalides CeHaVIO_4_ (Ha = Cl, Br; VI = Mo,
W). Dalton Trans..

[15] Dorn K. V., Schleid T., Hartenbach I. (2016). Tb_3_Cl_3_[WO_6_]: The Final Member in a Series of Lanthanoid­(III)
Chloride
Oxidotungstates­(VI) Bearing Trigonal Prismatic [WO_6_]^6–^ Units. Z. Anorg. Allg. Chem..

[16] Dorn K. V., Blaschkowski B., Förg K., Netzsch P., Höppe H. A., Hartenbach I. (2017). Prism Inside: Spectroscopic and Magnetic Properties
of the Lanthanide­(III) Chloride Oxidotungstates­(VI) *Ln*
_3_Cl_3_[WO_6_] (*Ln* =
La-Nd, Sm-Tb). Z. Anorg. Allg. Chem..

[17] Dorn K. V., Hartenbach I. (2019). Press to Success:
Gd_5_FW_3_O_16_—The First Gadolinium­(III)
Fluoride Oxidotungstate­(VI). Crystals.

[18] Lipp C., Schleid T. (2009). Die Selten-Erd-Metall­(III)-Fluorid-Oxoselenate­(IV)
MF­[SeO_3_] (M = Y, Ho–Lu) imYF­[SeO_3_]-Typ/
The Rare-Earth Metal­(III) Fluoride Oxoselenates­(IV) MF­[SeO_3_] (M = Y, Ho–Lu) with YF­[SeO_3_]-type Structure. Z. Naturforsch..

[19] Schleid T., Strobel S., Dorhout P. K., Nockemann P., Binnemans K., Hartenbach I. (2008). YF­[MoO_4_] and YCl­[MoO_4_]: Two Halide Derivatives of Yttrium
Ortho-Oxomolybdate -
Syntheses, Structures, and Luminescence Properties. Inorg. Chem..

[20] Schustereit T., Schleid T., Hartenbach I. (2014). Solvochemical Synthesis and Crystal
Structure of the Fluoride-Derivatized Early Lanthanoid­(III) *ortho*-Oxidomolybdates­(VI) *Ln*F­[MoO_4_] (*Ln* = Ce - Nd). Eur. J.
Inorg. Chem..

[21] Hartenbach I., Strobel S., Dorhout P. K., Schleid T. (2008). Crystal Structure,
Spectroscopic Properties, and Magnetic Behaviour of the Fluoride-Derivatized
Lanthanoid­(III) *Ortho*-Oxomolybdates­(VI) *Ln*F­[MoO_4_] (*Ln* = Sm-Tm). J. Solid State Chem..

[22] Link L., Niewa R. (2023). *Polynator*: a tool
to identify and quantitatively
evaluate polyhedra and other shapes in crystal structures. J. Appl. Crystallogr..

[23] Hoppe R. (1979). Effective
Coordination Numbers (ECoN) and Mean Fictive Ionic Radii (MEFIR). Z. Kristallogr..

[24] Nespolo M., Guillot B. (2016). CHARDI2015: charge
distribution analysis of non-molecular
structures. J. Appl. Crystallogr..

[25] Nespolo M. (2016). Charge distribution
as a tool to investigate structural details. IV. A new route to heteroligand
polyhedra. Acta Crystallogr..

[26] Hartenbach I., Höppe H. A., Kazmierczak K., Schleid T., Synthesis (2014). Synthesis, crystal structure and
spectroscopic properties of a novel yttrium­(iii) fluoride dimolybdate­(vi):
YFMo_2_O_7_. Dalton Trans..

[27] Müller S. L., Blaschkowski B., Hartenbach I. (2017). No Pyroanions: The Representatives
of the Lanthanoid­(III) Fluoride Oxidodimolybdates­(VI) *Ln*FMo_2_O_7_ with the Smaller Cations (*Ln* = Eu-Yb). J. Solid State Chem..

[28] Shannon R. D. (1976). Revised
Effective Ionic Radii and Systematic Studies of Interatomic Distances
in Halides and Chalcogenides. Acta Crystallogr..

[29] Busca G. (2002). Differentiation
of Monooxo and Polyoxo and of Monomeric and Polymeric Vanadate, Molybdate
and Tungstate Species in Metal Oxide Catalysts by IR and Raman Spectroscopy. J. Raman Spectrosc..

[30] Oreshonkov A. S., Roginskii E. M., Shestakov N. P., Gudim I. A., Temerov V. L., Nemtsev I. V., Molokeev M. S., Adichtchev S. V., Pugachev A. M., Denisenko Y. G., Structural (2020). Structural, Electronic and Vibrational
Properties of YAl_3_(BO_3_)_4_. Materials.

[31] Förster T., Ceccon L., Bendel B., Bettinelli M., Piccinelli F., Suta M. (2026). Analysis of the magnetic
dipolar
nonradiative decay of the ^5^D_1_ level of Eu^3+^ in single-crystalline huntite-type REAl_3_(BO_3_)_4_: Eu^3+^ (RE = Y, Gd, Lu). Mater. Adv..

[32] Kellendonk F., Blasse G. (1982). Luminescence and energy
transfer in TbAl_3_B_4_O_12_. J. Phys. Chem.
Solids.

[33] Vasquez S. O., Flint C. D., Sabry-Grant R. (1995). A shell model
for energy transfer
in lanthanide elpasolite crystals. J. Appl.
Spectrosc..

[34] Rabouw F.
T., den Hartog S. A., Senden T., Meijerink A. (2014). Photonic effects
on the Förster resonance energy transfer efficiency. Nat. Commun..

[35] Blasse, G. ; Grabmeier, B. C. Luminescent Materials; Springer-Verlag: Berlin, Heidelberg, 1994.

[36] Koziskova J., Hahn F., Richter J., Kožíšek J. (2016). Comparison
of different absorption corrections on the model structure of tetrakis­(μ^2^-acetato)-diaqua-di-copper­(II). Acta
Chim. Slovaca.

[37] Sheldrick G. M. (2008). A short
history of SHELX. Acta Crystallogr..

[38] Sheldrick G. M. (2015). Crystal
Structure Refinement with SHELXL. Acta Crystallogr..

[39] Rodriguez-Carvajal J. (1993). Recent advances
in magnetic structure determination by neutron powder diffraction. Phys. B.

[40] Bain G. A., Berry J. F. (2008). Diamagnetic Corrections and Pascal’s
Constants. J. Chem. Educ..

